# Unravelling Prokaryotic Codon Usage: Insights from Phylogeny, Influencing Factors and Pathogenicity

**DOI:** 10.2174/0113892029325491240919151045

**Published:** 2024-10-01

**Authors:** Ujwal Dahal, Anu Bansal

**Affiliations:** 1 Department of Biochemistry, School of Bioengineering and Biosciences, Lovely Professional University, Punjab 144411, India

**Keywords:** Prokaryotic codon usage, archaea, bacteria, phylogeny, codon usage software, pathogenic prokaryotes

## Abstract

Analyzing prokaryotic codon usage trends has become a crucial topic of study with significant ramifications for comprehending microbial genetics, classification, evolution, and the control of gene expression. This review study explores the numerous facets of prokaryotic codon usage patterns, looking at different parameters like habitat and lifestyle across broad groups of prokaryotes by emphasizing the role of codon reprogramming in adaptive strategies and its integration into systems biology. We also explored the numerous variables driving codon usage bias, including natural selection, mutation, horizontal gene transfer, codon-anticodon interaction, and genomic composition in prokaryotes through a thorough study of current literature. Furthermore, a special session on codon usage on pathogenic prokaryotes and the role of codon usage in the phylogeny of prokaryotes has been discussed. We also looked at the various software and indices that have been recently applied to prokaryotic genomes. The promising directions that lay ahead to map the future of codon usage research on prokaryotes have been emphasized. Codon usage variations across prokaryotic communities could be better understood by combining environmental, metagenomic, and system biology approaches.

## 1 INTRODUCTION

The codons are degenerate, as is widely known. Two to six synonymous codons can encode 18 of the 20 standard amino acids except methionine and tryptophan, both being encoded by the same genetic code. Codon usage bias (CUB), a phenomenon in which particular synonymous codons are preferred over others, has been discovered in all genomes examined [1]. CUB is present in many organisms and functions as a second genetic code inside the codons. The bias in the repetition of synonymous codons differs between genomes as well as within a single gene and among functionally related genes. The mechanisms that underlie codon usage bias across all forms of life are fascinating. CUB arises from mutations occurring in the second or third positions within specific genetic codes. This occurs when there is a substitution of one codon for another that encodes the same amino acid. Since these mutations do not alter the amino acid sequence of the protein, they are termed synonymous or silent mutations [2]. As a result, biased mutational patterns lead to codon usage bias, where certain codons may be retained through selection while others may be more susceptible to mutation [3]. The local recombination rate affects how genomic variation and GC-biased genes influence frequency of favored codons. Additionally, codon-anticodon interactions and codon usage bias in highly expressed genes being correlated to the specified quantity of particular tRNA, are equally significant in codon usage evolution in prokaryotes [4-6].

Similarly, the deliberate alternation of the genetic code usage within prokaryotes (codon reprogramming) plays a pivotal role in their adaptive strategies and interactions with genetic material, showcasing a dynamic response to environmental challenges and interspecies competition [7]. By strategically modulating codon usage, bacteria can enhance their efficiency and specificity, thereby bolstering their resistance to viral predation [3, 7]. Furthermore, in the context of horizontal gene transfer, prokaryotes employ codon reprogramming to facilitate the CRISPR-Cas (Clustered Regularly Interspaced Short Palindromic Repeats and CRISPR-associated proteins) defense mechanisms acquisition and integration of beneficial genetic material from other organisms [8]. Codon optimization of genes involved in antibiotic resistance or metabolic pathways can enhance the fitness of prokaryotes in challenging environments. This adaptive capability not only allows prokaryotes to thrive in diverse ecological niches but also underscores their capacity to evolve rapidly through genetic exchange [7, 8]. Therefore, the equilibrium between all these factors is what leads to the development of synonymous codon usage bias, which significantly promotes genome evolution [3, 6-8].

A significant portion of biological diversity on Earth consists of microscopic prokaryotic creatures. Millions of distinct species make up the domains Archaea and Bacteria. Prokaryotic variety evolved 3.8 billion years ago, which is 2 billion years longer than the history of eukaryotic creatures [9]. During the Hadean and Archaean Eons (before 2.5 billion years ago), characterized by extreme heat and volcanic activity, prokaryotes evolved mechanisms for survival in harsh environments. The Proterozoic Eon (2.5 billion to 5.4 million years ago) saw significant environmental shifts, including rising oxygen levels leading to the evolution of photosynthetic organisms like cyanobacteria. Furthermore, during Phanerozoic Eon (5.4 million years ago to the present day), prokaryotes continued to evolve alongside multicellular organisms, influenced by factors such as mutation rates, environmental pressures, ecological interactions like symbiosis and competition, and the widespread phenomenon of horizontal gene transfer [10, 11]. This extended evolutionary time is the cause of amazing prokaryotic diversity and a wide range of habitats. The prokaryotes are an essential part of the biosphere as they catalyze the biogeochemical cycles that maintain all life on Earth. As a result, they are the life- sustaining agents of the biosphere [12].

To date, more than 40000 (Archaea 5000 and Bacteria 35000) species have been fully characterized, and over 200000 bacterial and archaeal genomes have been sequenced and made available in public databases [10, 11]. These genomes, which are made up of many gene sets with different histories of origin and ancestry along with various expression tendencies constitute the living legacy of the oldest living beings on the planet. The evolutionary connections among many of the sequenced genomes are unknown, and a greater fraction of the deposited genomes remain unexplored. The query regarding how cellular units adapt their coding mechanisms to meet protein requirements, using synonymous codons in organisms with genomic GC composition normally between 20% and 80%, has been a long-standing issue. This question is highlighted by the genetic diversity observed in studies with incomplete or imprecise phylogenetic analyses [13].

All these queries could be solved by studying codon usage in prokaryotes by revealing evolutionary relationships influenced by mutational biases and natural selection, providing insights into shared genetic history, and complementing traditional phylogenetic markers. The study of codon usage in prokaryotes plays a pivotal role in modern genomics, bioinformatics, and evolutionary biology, particularly within the framework of systems biology. Codons, triplet nucleotide sequences that encode specific amino acids during protein synthesis, are not randomly distributed across genomes but exhibit distinct patterns influenced by various evolutionary and functional constraints. Bioinformatics approaches enable the analysis of codon usage biases across prokaryotes, shedding light on their adaptation strategies, metabolic efficiencies, and evolutionary histories. This integrative approach provides deeper insights into how genetic information is translated into functional proteins, influencing cellular processes and organismal fitness. By deciphering these codon usage patterns, researchers can uncover evolutionary trends, assess the impact of environmental factors on genomic evolution, and predict the expression levels of genes. Thus, codon usage analysis serves as a powerful tool in systems biology, bridging the gap between genotype and phenotype while elucidating the molecular basis of biological diversity and adaptation of prokaryotes [6, 14]. Here, we aim to summarize how prokaryotic organisms adjust codon usage and the reasons why certain biases worked for the advancement of prokaryotes in terms of phylogenic evolution, natural habitat and lifestyle, pathogenicity, and translational perspective. We also have discussed some special sections like codon usage trends in pathogenic prokaryotes.

## 2 PHYLOGENY AND ITS RELATIONSHIP WITH CODON USAGE

Determining the emergence of distinct prokaryotic lineages is a significant challenge due to the lack of reliable calibration points, specifically fossils, for prokaryotes. Nevertheless, initial examinations of prokaryotic phylogeny suggest an ancient origin spanning over 3 billion years. Pioneering research led by Carl Woese and colleagues extensively employed molecular sequencing techniques, particularly the analysis of 16S rRNA sequence data across a broad spectrum of species. This research brought to light an overlooked category of prokaryotes, initially labeled Archaebacteria and later rebranded as Archaea [15, 16]. This group encompasses methanogens, thermoacidophiles, and halophilic organisms, exhibiting a distinct phylogenetic separation from other prokaryotes, initially referred to as eubacteria and later renamed bacteria. Archaebacteria demonstrated a genetic proximity to both eukaryotes and eubacteria based on 16S rRNA data. This discovery, along with a range of other distinguishing features of Archaebacteria, prompted Woese and colleagues to propose Archaebacteria as a distinct phylum [16, 17]. These features include the absence of muramic acid in their cell walls, unique membrane lipids with ether-linked isoprenoid side chains (instead of diacyl esters found in other bacteria), distinct RNA polymerase subunit structures, and variations in sensitivity profiles to various antibiotics. Consequently, prokaryotes are dichotomized into two primary lines of descent such as Archaea and genuine bacteria.

The phylogeny of prokaryotes, which encompasses Archaea and Bacteria, reveals evolutionary relationships based on genetic similarities and divergence over time. CUB has been observed to correlate with phylogenetic topology in several ways, reflecting both shared ancestry and adaptive divergence among different taxa. At a broad phylogenetic scale, prokaryotic lineages that share a more recent common ancestor tend to exhibit similar patterns of codon usage. This similarity arises because closely related species inherit similar genomic compositions, including codon usage preferences, from their ancestors [18]. For instance, within the Firmicutes phylum, species such as *Bacillus subtilis* and * Clostridium difficile* show conserved codon preferences for certain amino acids like leucine and serine, reflecting their close evolutionary relationship and shared ecological niches. Consequently, phylogenetically related organisms often display comparable biases in codon usage due to their shared evolutionary history [ 19 ]. However, variations in codon usage bias can also occur between closely related taxa or within the same phylogenetic group. These variations may arise from adaptive evolution in response to different ecological niches, selective pressures, or specific genomic features unique to certain lineages. Environmental factors such as temperature, pH, and nutrient availability can influence codon preferences in bacterial genomes, leading to divergent codon usage patterns even among closely related species [20]. For example, *Escherichia coli* and *Salmonella enterica*, both members of the Enterobacteriaceae family, demonstrate distinct codon usage patterns influenced by their respective lifestyles and environmental adaptations*. E. coli*, commonly found in the human gut, exhibits biased codon usage favoring codons optimized for efficient translation under nutrient-rich conditions, whereas *S. enterica*, which can cause salmonellosis in various hosts, shows adaptations for survival in diverse host environments [20, 21].

Moreover, horizontal gene transfer (HGT) plays a significant role in shaping codon usage bias across phylogenetic boundaries in prokaryotes. Genes acquired through HGT often retain their original codon usage patterns from their donor organisms, contributing to mosaic genomes with heterogeneous codon biases within a single species or population. This phenomenon blurs strict phylogenetic correlations in codon usage and underscores the dynamic nature of genomic evolution in prokaryotes [12, 22]. For instance, genes involved in antibiotic resistance in *Staphylococcus aureus *may display codon biases reflective of their origins in other bacterial species, illustrating how HGT shapes codon usage independently of strict phylogenetic relationships [22].

In summary, while codon usage bias generally reflects phylogenetic relationships among prokaryotes, its patterns can also diverge due to adaptive evolution and HGT. These insights underscore the dynamic interplay between genomic evolution, environmental adaptation, and the complex patterns of codon usage bias in shaping the diversity and evolutionary trajectories of prokaryotic life [12, 22].

## 3 CODON USAGE IN PROKARYOTES

Codon usage in prokaryotes is a fundamental aspect of genetic information processing that influences protein synthesis efficiency, fidelity, and adaptation. The genetic code is degenerate, with most amino acids encoded by multiple synonymous codons. However, prokaryotic genomes often exhibit biased usage of these codons, where certain synonymous codons are preferred over others. Multiple studies have demonstrated that translational selection, affected by parameters including abundance tRNA [23, 24], environmental adaptations [12], and genomic GC content [25], plays a vital role in defining codon usage patterns in prokaryotes.

Codon usage bias in prokaryotes is intricately linked to environmental adaptations, including temperature tolerance and nutrient acquisition, which are often reflected in genomic GC content [25]. GC-rich genomes tend to prevail in thermophilic bacteria and archaea, where higher GC content contributes to DNA stability at elevated temperatures [26]. These organisms typically exhibit codon bias favoring GC-rich codons, which encode amino acids that stabilize proteins under extreme thermal conditions. Conversely, GC-poor genomes are common in mesophiles and psychrophiles adapted to moderate or cold environments, where lower GC content may confer advantages such as enhanced DNA flexibility and reduced energy costs during replication and transcription. Moreover, Prokaryotes thriving in nutrient-rich environments often prioritize codons recognized by abundant tRNAs, promoting efficient translation and high protein yields. In contrast, microbes adapted to nutrient-poor conditions may exhibit codon bias that conserves energy by favoring less costly codons during protein synthesis [26, 27].

Different species have different preferred codon choices depending on their tRNA repertoire and other relevant parameters [4, 5]. As per the "mutation-selection-drift model" random genetic drift, natural selection, and mutation work together to produce genetic variation within prokaryotes due to which prokaryote genomes show a wide range of GC content. This model balances bias through selection-based restrictions, altering GC content and codon usage. There are variations in the intragenomic codon usage of singletons and core genes in different gene sets. Selection factors, gene function, and cellular effects lead to variances in genetic code utilization within a single cell. Optimal codon-anticodon interactions, determined by the amount of a certain tRNA, correspond to the biased utilization of genetic code in prokaryotes, nonstandard codon-anticodon interactions have an effect on the development of genetic codes. Although translational selection is present in most prokaryotes, different genes and deciding factors have different levels of codon usage bias [28, 29]. The predominance of translational selection is indicated by some species, such as *Alkaliphilus metalliredigens* [30] and *Escherichia coli* [31], which exhibit a positive association between codon bias and protein levels. Others, like as *Helicobacter pylori* [32], show little variation in the utilization of synonymous codons, which is explained by translational selection that is weak and mutation. The correlation between phenotypic features in many prokaryotes and codon usage bias is demonstrated by the comprehensive effect of highly adapted codons on cellular processes [33].

The various factors affecting codon usage trends in prokaryotes have been illustrated in Fig. (**1**) and the role of codon usage bias in various prokaryotes has been detailed in Table **1**. For instance, *Pseudomonas aeruginosa* utilizes specific codons in biofilm formation and antibiotic resistance, optimizing adaptability in various host environments [34]. *Salmonella enterica* exploits codon preferences for survival and colonization within the intestinal tract [35], while *Mycobacterium tuberculosis* exhibits CUB patterns influenced by HGT in adapting to the human host and evolving virulence traits [36]. Cyanobacteria, such as *Synechococcus elongates*, show specific codon preferences in genes related to photosynthesis, enhancing efficiency in protein synthesis that is crucial for their metabolic pathways [37]. Archaeal species like *Haloferax volcanii* align codon usage with the abundance of tRNAs to thrive in hypersaline environments [38].

Examining the diversity of genetic code usage in prokaryotic genomes and genes has been done in a variety of ways. Grantham and colleagues calculated the first list of codon usage frequencies in 1980 using mRNA sequences with 51 or more genetic codes. Since the 1980s, a wide range of software and indices have been created to define, examine, and quantitatively estimate genetic codes use bias or genetic code usage preferences. Several software tools, such as GeneMark [58], CAIcal [59], EMBOSSCusp [60], and CodonW [55], have been utilized for evaluating codon usage preferences, with many focusing on calculating indices like Codon Adaptation Index (CAI), Effective Number of Codons (ENC), and others.

The indices used for analyzing genetic code usage bias in prokaryotes have been categorized as the indices that examine the observed genetic codes use distribution of the targeted set of genetic codes against a mentioned set of highly-represented genes, that examine data based on the supposition of equal usage of codons coding same amino acid, indices dependent on the adaptation of tRNA levels and their supply, indices dependent on complex trends of genetic code usage and also indices dependent on direct experiment analysis of translational as well as transcriptional elongation [2, 61]. Popular most common indices applied in the examination of genetic code usage bias is CAI developed by Sharp and Li in 1987 followed by ENC developed by Wright 1990 and Relative Synonymous Codon Usage (RSCU) developed by Sharp *et al*. in 1986 which can be used to distinguish chromosome and plasmid in bacteria [62a and b, 63a and b]. Other commonly used indices include Frequency of optimal codons, correspondence analysis, Neutrality plot, Parity bias plot analysis, Relative codon adaptation, Codon deviation coefficient, Relative codon deoptimisation index, Synonymous codon usage order, GRAVY, and AROMO analysis [51, 64-66]. The list of the various CUB software and indices that have been used in prokaryotes has been illustrated in Fig. (**2**).

## 4 FACTORS AFFECTING CODON USAGE TRENDS IN PROKARYOTES

### 4.1 Genomic Features

Genomic features, such as GC content, gene length, and level of gene expression, significantly influence codon usage bias in prokaryotic organisms, reflecting adaptations to environmental conditions and evolutionary histories. However, it is to be noted that the correlation between genomic features and codon usage trends highlights their association rather than causation, emphasizing the intricate interplay between genetic features and codon selection in prokaryotic organisms [ 3, 74].

The influence of genomic composition on codon usage bias is important in prokaryotes, as there is considerable variation in the frequency of individual codons. The amount of guanine (G) and cytosine (C) bases in DNA is known as the GC content, and it is one important genomic characteristic that affects codon usage. Prokaryotic genomes with high GC contents typically have biased codon usage patterns because of their propensity for codons rich in G and C. The thermodynamic stability of G-C base pairs, which supports the general stability of the mRNA secondary structure, is frequently blamed for this bias [75]. Therefore, prokaryotes with a high GC content would prefer codons that start with G and C, which would indicate that their genomes are able to adapt to different environmental situations through enhanced protein stability, regulatory capabilities, and evolutionary flexibility. For instance: *Mycobacterium tuberculosis* favors G and C nucleotides across the codon due to its high GC content [36].

Gene length is another genetic characteristic that influences codon usage bias. Shorter genes are typically linked to improved translational efficiency and expression levels in prokaryotes. The "translational selection hypothesis," which explains this phenomenon, postulates that codon usage influences natural selection in a way that maximizes translation accuracy and speed. Stronger selection pressure for effective translation may be applied to genes, which might result in biased codon usage that speeds up and improves the accuracy of protein synthesis [76]. The best example can be *B. subtilis* [56] in which longer genes have a stronger preference for specific synonymous codons. The availability of tRNAs and other elements that affect translation efficiency determines the particular codons that are preferred in the species.

Bacterial genomes frequently show differences in the bias in codon usage across genes that are highly and lowly expressed. Stronger bias towards optimal codons, which are effectively recognized by an abundance of tRNAs, is more likely to be seen in highly expressed genes, highlighting the relationship between codon selection and gene expression levels. *Caulobacter crescentus* displays CUB in cell cycle-related genes (highly expressed) to contribute efficient and accurate protein synthesis [45].

### 4.2 Environment

The environment has a significant impact on the codon usage patterns of prokaryotic species, which are indicative of the adaptive methods these organisms use to survive in a variety of settings. Arella and group in 2021 suggested that prokaryotes with specific physical characteristics and residing across comparable living conditions share similar genetic code preferences [6]. One important environmental element that influences codon usage is temperature; prokaryotes show specialized preferences for codons that are adapted to particular temperature ranges. Due to their greater energy favorability at higher temperatures, codons rich in G and C are frequently preferred by thermophilic bacteria, which are adapted to hot temperatures. Additionally, bacteria that are psychrophiles show a predilection for codons that include G and C. These nucleotides help to stabilize DNA and RNA structures as temperatures rise. The relationship between codon usage and temperature emphasizes how environmental adaptation alters the genetic code to maximise cellular functioning across a range of temperatures [77].

Other environmental conditions that affect codon usage patterns in bacterial genomes are salt and pH. Bacteria known as extremophiles, which flourish in environments of severe salinity or pH, frequently have certain codon biases to improve their adaptability. Acidophiles like *Acidithiobacilus ferrooxidans* could choose codons with a greater GC content, which would help keep their genetic material stable in an acidic environment [48]. *Halobacterium salinarum* is acclimated to high salinities and exhibit codon preferences that are indicative of the necessity of effective protein synthesis in salt settings [49]. Because of these modifications in codon usage, prokaryotic organisms may survive in conditions with severe pH or salinity while still maintaining optimal cellular processes like protein folding and stability. The complex relationship that exists between codon usage and environmental factors highlights the role that genetic flexibility plays in bacterial evolution [7].

Moreover, codon usage patterns of bacterial genomes can be influenced by the nutrients that are available in the surrounding environment. Prokaryotes, for example, may show a preference for codons that promote fast translation in nutrient-rich settings, maximizing the use of available resources for development and reproduction. On the other hand, distinct codon preferences may result from the selection pressure for effective resource utilization in nutrient-poor settings. The dynamic nature of codon usage patterns reflects the resilience of prokaryotic genomes on changing nutritional circumstances and shows how environmental variables aid in the expression of the genetic code in response to ecological niches [78].

### 4.3 Functional Constraints

The usage of codons is subjected to functional constraints, which significantly influence the genetic code composition of organisms. The association between codons and the amino acids they encode is the main functional restriction. One or more codons are responsible for encoding each amino acid; synonymous codons denote distinct nucleotide triplets that share the same amino acid coding. These synonymous codons can show varied usage frequency despite unchanged amino acid sequences in a protein. Therefore it is suggested that the requirement to preserve appropriate protein folding, stability, and functionality gives rise to functional limitations [63]. Because they affect the precision and speed of translation, some codons may be more advantageous than others, which in turn affects the shape and function of proteins. In *Mycobacterium tuberculosis*, overrepresentation of the alanine-coding codon GCG supports the stability of mRNA secondary structures and is thought to represent a response to the host environment [36].

Another important component that significantly contributes to codon usage bias is translational efficiency. The amount of transfer RNA (tRNA) molecules in the cellular environment affect how quickly and accurately translation proceeds and differing amounts of tRNAs can recognize different codons. Rich tRNA-corresponding codons are frequently preferred because they promote quicker translation and lower the risk of mistakes. Natural selection optimizes translation rates by acting on codon usage patterns; this process is referred to as translational selection. The relationship between translational efficiency and codon usage emphasizes how crucial it is to strike a balance between correct amino acid incorporation and quick protein synthesis during translation [48, 79].

### 4.4 tRNA Abundance and Availability

The preference for particular codons in bacterial genomes is mostly determined by the amount of transfer RNA (tRNA) molecules. A particular amino acid is transported to the ribosome by each tRNA during translation, and the quantity of these tRNAs differs between species and even within cellular environments. Translational selection is the tendency of prokaryotic organisms to choose codons that correlate to high levels of tRNAs. a high quantity of a particular tRNA facilitates precise and efficient translation by allowing the correct codons to be quickly identified and matched with the right amino acids. This inclination towards codons linked to high tRNA abundances is indicative of an adaptive tactics to maximise translational efficiency and lower the risk of mistakes during protein synthesis. Genes involved in secondary metabolite biosynthesis often exhibit codon usage bias correlated with tRNA availability in prokaryotes like *Streptomyces coelicolor* [48].

Furthermore, codon usage is subject to functional restrictions that go beyond the translation process itself. RNA secondary structures and binding sites for RNA-binding proteins are examples of regulatory factors that can affect the choice of particular codons within mRNA sequences. The functional consequences of codon selection are further highlighted by the fact that these components can impact mRNA stability, localization, and interactions with cellular machinery [2]. Genes encoding heat-shock proteins prefer codons that contribute to the stability of mRNA secondary structures in *Thermus thermophiles* which ultimately helps in protein folding at elevated temperature [34, 80].

In the context of codon families, the link between tRNA availability and codon usage bias is clear. Codon families are groups of synonymous codons with different nucleotide sequences that code for the same amino acid. Because related tRNAs are abundant in some codon families, some codons may be utilised more frequently than others within that family. Within these groups, prokaryotic genomes frequently display a biased use of synonymous codons, such as *Pseudomans aeruginosa* which reflects the varying availability of tRNAs. This tendency is more noticeable in highly expressed genes because there is greater selection pressure to translate a gene efficiently. The complex interaction between codon preference and tRNA abundance highlights how translational selection shapes the codon usage patterns seen in bacterial genomes [33, 81].

Furthermore, during evolution and adaptation, variations in tRNA abundance have shown an effect on codon usage patterns of prokaryotes like *E. coli* [82]. The cellular pool of tRNAs may change when prokaryotes experience different environmental circumstances in order to maximize protein production. A prokaryote may, for example, adjust the abundance of particular tRNAs to match codon preferences that support effective translation of genes involved in adjusting to the new conditions if it comes into contact with an environment that has a change in the availability of nutrients. The dynamic correlation between tRNA availability and codon usage highlights the functional relevance of the interaction between tRNA abundance and codon choice, reflecting the resilience of bacterial genomes to shifting selection forces. Overall, the complex mechanisms governing translational efficiency and codon usage in bacterial genomes are highlighted by the significance of tRNA abundance in these processes and environmental effect [83].

### 4.5 Horizontal Gene Transfer

In prokaryotic evolution, HGT is a ubiquitous mechanism that permits the acquisition of genetic material from unrelated species. The patterns of codon usage in bacterial genomes can be greatly impacted by this gene transfer. Foreign genes may have different codon preferences from the receiving organism when they are introduced by HGT. The integrated genes may therefore first show a bias in codon usage that is not compatible with the host genome. HGT is the process by which codon usage of acquired genes eventually aligns with the recipient genome. Selective pressures to maximize translational efficiency and protein folding are what propel HGT, which in turn helps to successfully integrate and express the foreign genes within the host organism [84, 85].

The effects of HGT on codon usage are seen in parts of bacterial genomes linked to mobile genetic components like phages and plasmids. These components frequently act as carriers of new genetic material into prokaryotic communities through horizontal gene transfer. In order to conform to the preferences of the receiving organism, the genes carried by these components may alter their codon usage, enabling effective expression and functional integration [86]. As a result, the dynamic and adaptable character of codon usage patterns in these microbes is partly due to the mosaic structure of prokaryotic genomes, which is the outcome of the introduction of foreign genetic material through HGT. HGT in Cyanobacterium helps them to adapt to the host environment and optimize protein synthesis [37].

The codon usage bias of particular functional gene categories is also shaped by horizontal gene transfer. Because precise control and coordination of these fundamental functions are required, genes involved in translation, transcription, and replication are frequently more conserved in terms of codon usage. On the other hand, because of the effect of horizontally transmitted genes, genes linked to adaptive functions similar to those engaged in environmental response or niche-specific metabolic pathway show more diversity in codon usage. Species like *Vibrio parahaemolyticus* [43], *Mycobacterium avium* [36] and *Bacillus anthracis* [56] acquire distinct genomic islands through HGT and there is significant variation in genomic composition of horizontally acquired genes to adapt in host environment. This capacity to quickly adapt to new ecological niches and environmental obstacles is made possible by the integration of foreign genes, which enhances the overall diversity and adaptability of prokaryotic genomes [85].

### 4.6 Codon-anticodon Interaction

Codon- anticodon interaction is one of the crucial factor that effects codon usage in prokaryotes. The interaction between codons and anticodons is essential for the formation of proteins during translation in prokaryotic organisms. On the mRNA, the codon designates a specific amino acid or a start/ stop signal. Complementary to the codon is the anticodon, a three-nucleotide sequence on a tRNA molecule. The relevant amino acid is transported by the tRNA to the ribosome, the site of protein synthesis. The precise alignment of amino acids in the developing polypeptide chain is guaranteed by the specificity of the codon-anticodon interaction causing codon usage bias in prokaryotes [87, 88].

The stability and specificity of the codon anticodon interaction is a key factor in determining the fidelity of translation and preventing codon reprogramming in prokaryotes [89, 90]. Typically, the codon anticodon interaction is stabilized by hydrogen bonds, with the optimal interaction energy being a balance between maximizing binding strength and minimizing the risk of mismatches [91-93]. However, in certain cases, prokaryotes have evolved mechanisms to reprogram the genetic code by altering the standard codon-anticodon interactions [90]. For example, in some bacteria, the anticodon of the tRNA that recognizes the stop codon UGA can be modified to pair with the amino acid selenocysteine instead of triggering translation termination. This allows the UGA codon to specify selenocysteine incorporation, rather than acting as a stop signal. Such codon reprogramming events are crucial for the expression of specialized proteins in prokaryotes [89, 90, 94].

**Fig. (3):**
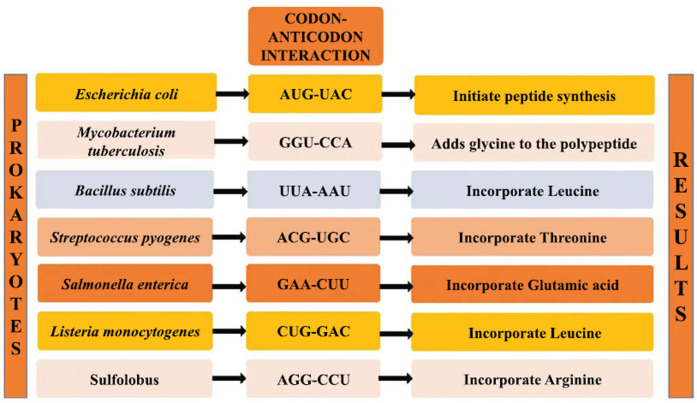
Codon-anticodon interaction in prokaryotes [87, 88, 95-100].

In prokaryotes like *E. coli* , *M. tuberculosis* and *B. subtilis* , interaction between codons on mRNA and anticodons on tRNA is pivotal for the precise translation of genetic information into proteins (Fig. **3**). Each mRNA codon specifies a particular amino acid, and tRNA molecules carrying complementary anticodons recognize and bind to these codons through base pairing during protein synthesis [90, 95]. This process occurs in coordination with ribosomes, where specific amino acids like glycine and leucine are sequentially added to the growing polypeptide chain in *M. tuberculosis* and *B. subtilis* respectively. In case of *E. coli*, codon anticodon interaction (AUG-UAC) initiate the synthesis of peptide chain (Fig. **3**). CUB in these prokaryotes reflects the preferential usage of certain specific synonymous codons over others, influenced by factors such as translation efficiency, accuracy, and the availability of corresponding tRNAs [90, 96-99, 101]. The codon-anticodon preference in various prokaryotic organisms for incorporation of specific amino acids are illustrated in Fig. (**3**).

## 5 CODON USAGE BIAS IN PATHOGENIC PROKARYOTES

In pathogenic prokaryotes, CUB is a complex phenomenon with broad implications for comprehension of the complexities of host-pathogen relationships, adaptive evolution, and possible therapeutic intervention uses. Deciphering codon usage bias offers a path toward discovering critical genes and possible targets for drugs in the context of vaccines and antibacterial treatments. Furthermore, pathogen-specific codon preferences can be used in vaccine design to generate strong immune responses. To maximise antigen expression and immunogenicity, for instance, the pathogen's codon usage patterns are taken into consideration during the design of vaccines against *Neisseria meningitides* [101].

A variety of variables affect the complex patterns of codon usage that are revealed by analyzing the genomes of pathogenic bacteria and archaea. In the opportunistic pathogen *Pseudomonas aeruginosa*, for example, genes linked to important processes show a translational advantage by favoring codons identified by numerous tRNAs. It is thought that this optimization helps the bacteria adapt to a variety of host settings [67]. In pathogenic prokaryotes, environmental adaptability is also crucial to the dynamics of codon usage. The gastrointestinal pathogen *Salmonella enterica* has unique codon usage preferences related to its adaptability to the intestinal environment of its host. The relationship between microbial genomes and their ecological niches is shown by these adaptations, highlighting the function of CUB as an evolutionary strategy for survival and growth [12, 38].

Moreover, it is remarkable how HGT affects codon usage patterns across pathogenic prokaryotes. Different sources of genes are frequently acquired by pathogenic prokaryotes, resulting in differences in codon preferences. The tuberculosis-causing agent, *Mycobacterium tuberculosis*, has codon usage patterns that are impacted by HGT events, which is indicative of the pathogens' complicated evolutionary past [36, 84-86]. Additionally, it is possible to comprehend the functional importance of codon usage bias in pathogenic prokaryotes by considering its involvement in virulence modulation. A causative agent of significant respiratory infection, *Streptococcus pneumoniae* exhibits codon usage patterns linked to genes involved in immune evasion and host colonization [102]. The complex connection that exists between CUB and virulence highlights the possibility of using pathogen specific codon usage as a target for medicinal therapies [103].

## 6 THE FUTURE OF PROKARYOTIC CODON USAGE STUDIES

Codon usage patterns are very crucial to be analyzed in order to interpret the genetic codes and to predict gene expression. Codon usage studies have a very wide-ranging scope in several disciplines, including molecular biology and synthetic biology, evolutionary biology, and pharmacology. Research on the analysis of codon usage has already shed light on genetic code, the control of gene expression, and the evolutionary trends not only in prokaryotes but also in other types of organisms. However, there are a number of intriguing directions for future research that can give new insights across all forms of life available on the planet which are summarized in Table **2**. Researchers can gain a deeper understanding of how prokaryotes use codons and adapt their translational machinery to various environmental conditions by leveraging developments in genomic technologies, comparative genomics, metagenomics, functional context integration, longitudinal studies, mobile genetic element analysis, synthetic biology, and multiomics approaches. Codon usage research will be essential in resolving the complexities of gene expression and evolution in prokaryotic organisms as the science develops [3, 104].

## CONCLUSION

This review study has provided a thorough investigation of prokaryotic codon usage, revealing its significance to both fundamental research and real-world applications. The processes behind the distinctive genetic adaptations of prokaryotes have been clarified by studying the patterns of codon usage in these organisms. Understanding the evolutionary constraints that have affected codon preferences across prokaryotes has become easier due to the addition of phylogenetic information. Furthermore, the relationship between prokaryotic environment and lifestyle and codon bias opens up new research directions for understanding the ecological and evolutionary factors that drove genetic development. The direct influence of codon usage bias on the pathogenicity of pathogenic prokaryotes adds more value to this topic. Therefore, we want to motivate academics to overcome the remaining issues and launch novel investigations by providing insights into the future directions and constraints of codon usage studies.

Overall, this review summarizes the major advancements achieved in codon usage research, highlighting its complexity and broad range of possible applications. This review has suggested the foundation for future investigations that will definitely contribute to continuously expanding prokaryotic studies, from comprehending the complicated genetic language of prokaryotes to using this information for biotechnological and medical breakthroughs.

## AUTHORS' CONTRIBUTIONS

UD: Writing- Original draft, Data curation, Conceptualization, Formal analysis, Investigation Validation. AB: Data curation, Formal analysis, Validation, Writing – review & editing, Conceptualization and Supervision.

## Figures and Tables

**Fig. (1):**
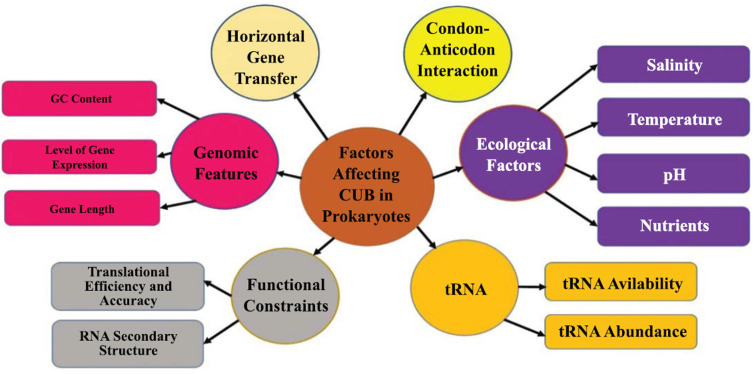
Factors affecting codon usage trends in prokaryotes [1-6, 52, 55, 57].

**Fig. (2):**
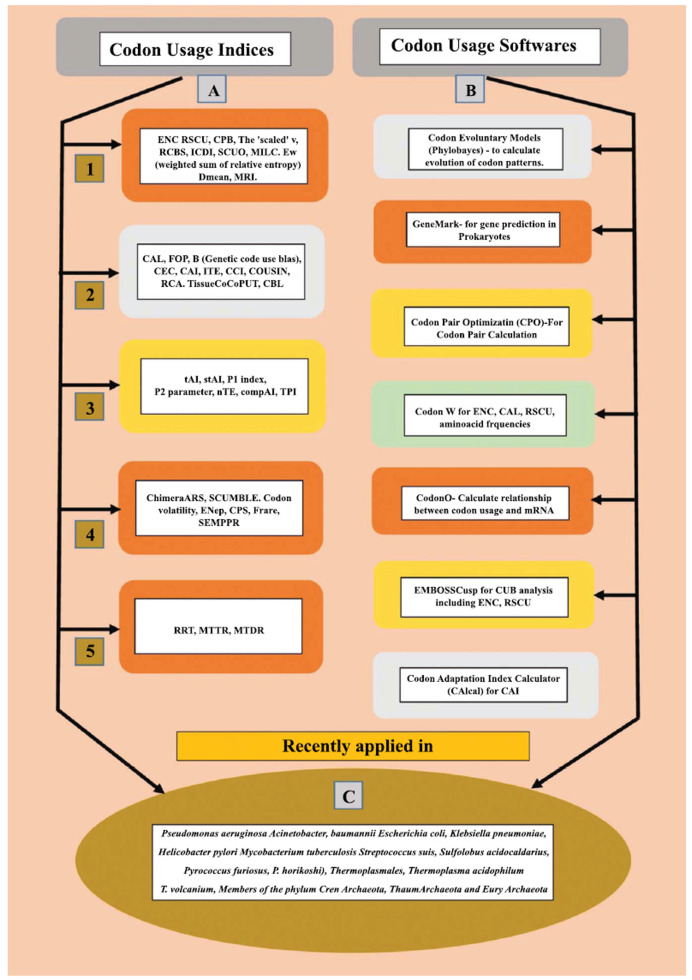
Codon usage software and indices used to study CUB in prokaryotic organisms. (**A**) Codon usage indices dependent on [62-66]. 1) Unequal use of synonymous genetic codes. 2) Codon frequency across a mentioned set of genes. 3) Adaptation to tRNA pool including supply. 4) Patterns of codon usage. 5) Laboratory analysis of translational and transcriptional elongation. (**B**) Codon usage softwares [55, 58-60, 67]. (**C**) Prokaryotes in which CUB has been recently studied [41, 42, 45, 48, 49, 68-73]. **Abbreviations:** Mean typical decoding rate (MTDR), Ribosome Residence Time (RRT), Mean typical transcription elongation rate (MTTR), Synonymous codon usage bias maximum-likelihood estimation (SCUMBLE), Effective number of codon-pairs (ENcp), Codon pair score (CPS), Frequency of rare codons (Frare), Stochastic evolutionary model of protein production rate (SEMPPR), tRNA adaptation index (tAI), Species-specific tRNA adaptation index (stAI), Normalized translational efficiency (nTE), Competition Adaptation Index (compAI), tRNA-pairing index (TPI), Codon adaptation index (CAI), Frequency of optimal codons (FOP), Codon bias index (CBI), Codon-enrichment correlation (CEC), Relative codon adaptation index (rCAI), Index of translation elongation (ITE), Self-Consistent Codon Index (CCI), Codon usage similarity index (COUSIN), Relative codon adaptation (RCA), Tissue specific Codon and Codon-Pair Usage Tables (TissueCoCoPUT), Effective number of codons (ENC), Relative synonymous codon usage (RSCU), Codon preference bias (CPB), Relative codon bias strength (RCBS), Intrinsic codon deviation index (ICDI), Synonymous codon usage order (SCUO), Measure independent of length and composition (MILC), Weighted sum of relative entropy (Ew), Mean dissimilarity based index (Dmean), and Mutational Response Index (MRI).

**Table 1:** Role of CUB in various prokaryotes.

**Prokaryotic Organism**	**Role of Codon Usage Bias**	**Findings from Codon Usage Studies**	**References**
*Pseudomonas aeruginosa*	Contribute to the pathogen's adaptability in diverse host environments	Biofilm formation and antibiotic resistance genes preferentially use codons corresponding to abundant tRNAs	[34]
*Salmonella enterica*	Involved in host colonization and survival within the intestinal environment	Exploit specific codons that align with the host environment	[35]
*Mycobacterium tuberculosis*	Adaptation to the human host and the evolution of virulence traits	CUB patterns influenced by the acquisition of genes through HGT	[36]
*Neisseria meningitidis*	Organization of antigen expression, ensuring the elicitation of robust immune response against bacterium	Exhibits specific codon preferences can be used in rational vaccine design	[39]
Cyanobacterium	Involve complex biochemical pathways with specific codon preferences optimized for efficient protein synthesis	HGT from another Cyanobacterium	[40]
*Haloferax volcannii(Archaeon)*	Helps to thrive in hypersaline environments	Codon usage aligns with the abundance of tRNAs	[38]
*Escherichia. coli*	Enhance translational efficiency	Codons for amino acids like Glycine (GGC and GGG) correspond to highly abundant tRNAs	[31]
*Bacillus subtilis*	Enhance translational efficiency	Genes encoding ribosomal proteins and components of the translation machinery tend to prefer codons corresponding to highly abundant tRNAs	[23]
*Thermus thermophilus*	Proper folding of transcripts at elevated temperature	Genes encoding heat-shock proteins prefer codons that contribute to the stability of mRNA secondary structures	[39]
*Mycoplasma pneumonia*	Reduction in overall genome size	Exhibits CUB associated with functional constraints related to genome compactness	[41]
*Synechococcus elongates (Photosynthesis gene)*	Ensuring rapid and accurate translation of proteins involved in photosynthesis	Photosynthetic exhibit a bias towards codons corresponding to highly abundant tRNAs	[37]
*Vibrio cholera*	Changes in codon usage patterns when transitioning from environment reservoirs to the human host	Genes encoding toxins and adhesion proteins, show adaptation in codon usage to optimize expression during infection.	[42]
*Vibrio parahaemolyticus*	Distinct genomic islands are acquired through HGT	Variations in genomic composition, particularly in horizontally acquired regions	[43]
*Thermococcus kodakarensis* (archaeon)	Enhance the stability of mRNA secondary structures, ensuring proper translation at high temperatures	Genes exhibit a preference for codons that end with G or C	[44]
*Caulobacter crescentus*	Contribute to efficient and accurate protein synthesis, especially in highly expressed genes	Displays CUB in cell cycle-related genes, which is linked to tRNA availability	[45]
*Streptomyces coelicolor*	Efficient translation of complex biosynthetic pathways	Genes involved in secondary metabolite biosynthesis often exhibit codon usage bias correlated with tRNA availability	[46]
*Psychrobacter arcticus*	Function optimally at low temperatures	Favoring codons that match the availability of cold adapted tRNAs	[47]
*Acidithiobacillus ferrooxidans*	Be adapted to a highly acidic pH environment	Reflect adaptations to optimize translational efficiency	[48]
*Alkaliphilus metalliredigens*	Adaptation to an alkaline environment	Reflect adaptations to optimize translational efficiency	[30]
*Halobacterium salinarum* (arhaeon)	Adapted to high salinity environment	The codon usage patterns in the genome are influenced by the need for efficient translation in a high salt environment	[49]
*Desulfovibrio desulfuricans*	Helps to thrive in the absence of oxygen in its environment	Influences codon usage patterns, optimizing translation under anaerobic conditions	[50]
*Clostridium thermocellum*	Optimizing translational efficiency in the niche	Codon usage reflects adaptations to both high temperature and anaerobic conditions	[51]
*Nitrosomonas europaea*	Helps to thrive in high concentrations of ammonia	The concentration of ammonia influences codon usage patterns, optimizing translational efficiency	[52]
*Geobacter sulfurreducens*	Helps in the biodegradation of radioactive metals	The redox potential of its environment influences codon usage in genes involved in the metal reduction and electron transfer process	[53]
*Synechocystis sp.*	Helps in the process of photosynthesis	Light intensity and photoperiod influence codon usage patterns in genes related to photosynthesis	[54]
76 different species of *Clostridium*	Clustering of pathogenic species	Pathogens use amino acids with lower biosynthetic cost	[55]
*Mycobacterium avium*	Contribute to virulence	Acquisition of pathogenicity islands	[36]
*Bacillus anthracis*	Contribute to virulence	Acquiring plasmids carrying toxin genes	[56]

**Table 2:** Future direction on the codon usage research on prokaryotes.

**S. No.**	**Future Direction**	**Possibilities**	**References**
1.	Analyzing comparative geno-mic signatures of prokaryotes	Can find conserved tendencies, species-specific adaptations, and gain better insights into the processes influencing codon usage biases	[2, 104]
2.	Analyzing codon usage bias in mobile genetic elements of prokaryotes	Genes, transfer of plasmid and phages between various prokaryotic species, and evolution can be better understood by understanding the codon usage biases in those elements. Additionally, examining the codon usage of mobile genetic elements can provide insight into the element's interaction with the translational apparatus of the host and impact the prokaryotic host's fitness	[3, 6]
3.	Metagenomic and environmental research in prokaryotes	Possible to learn more about the genetic potential and expression methods of uncultured prokaryotes by examining the codon usage in environmental samples	[78]
4.	Contextualization of function	CUB patterns can be examined in relation to gene function, expression levels, and protein characteristics to deduce better insights into selection forces influencing trends of codon usage. These integrative methods can aid in identifying genes susceptible to strong selection and those that have undergone particular environmental adaptation as well	[105]
5.	Prokaryotes in genetic engineering and synthetic biology	Researchers can optimize protein expression levels, raise protein production, and create more effective genetic constructs by modifying codon usage patterns in prokaryotic genes	[106]
6.	Bringing multi-omics data of prokaryotes together	Examine how codon usage affects gene expression, protein abundance, and metabolic pathways using this integrated method. It would also aid in identifying possible relationships between codon usage biases and post-transcriptional and post-translational regulatory mechanisms	[107]
7.	Longitudinal studies in prokaryotes	Can show how the dynamics of codon usage modifications respond to alterations in the environment, population constraints, and selection pressures	[108]

## LIST OF ABBREVIATIONS

AROMO: Aromaticity
CUB: Codon Usage Bias
GRAVY: Grand Average of Hydropathy
HGT: Horizontal Gene Transfer
mRNA: messenger RNA
tRNA: transfer RNA
